# The Role of SwrA, DegU and P_D3_ in *fla/che* Expression in *B. subtilis*

**DOI:** 10.1371/journal.pone.0085065

**Published:** 2013-12-27

**Authors:** Serena Mordini, Cecilia Osera, Simone Marini, Francesco Scavone, Riccardo Bellazzi, Alessandro Galizzi, Cinzia Calvio

**Affiliations:** 1 Department of Biology and Biotechnology, Università degli Studi di, Pavia, Pavia, Italy; 2 Department of Electrical, Computer and Biomedical Engineering, Università degli Studi di, Pavia, Pavia, Italy; University of Groningen, Groningen Institute for Biomolecular Sciences and Biotechnology, Netherlands

## Abstract

In *B. subtilis* swarming and robust swimming motility require the positive trigger of SwrA on *fla/che* operon expression. Despite having an essential and specific activity, how SwrA executes this task has remained elusive thus far. We demonstrate here that SwrA acts at the main σ^A^-dependent *fla/che* promoter P_A(*fla/che*)_ through DegU. Electrophoretic mobility shift assays (EMSA) reveal that SwrA forms a complex with the phosphorylated form of DegU (DegU~P) at P_A(*fla/che*)_ while it is unable to do so with either unphosphorylated DegU or the DegU32(Hy) mutant protein. Motility assays show that a highly phosphorylated DegU is not detrimental for flagellar motility provided that SwrA is present; however, DegU~P represses P_A(*fla/che*)_ in the absence of SwrA. Overall, our data support a model in which DegU~P is a dual regulator, acting either as a repressor when alone or as a positive regulator of P_A(*fla/che*)_ when combined with SwrA. Finally, we demonstrate that the σ^D^-dependent P_D3(*fla/che*)_ promoter plays an important role in motility, representing a contingent feedback loop necessary to maintain basal motility when *swrA* is switched to the non-functional *swrA* ^-^ status.

## Introduction

Regulation of flagellar motility in *Bacillus subtilis* appears to be exerted at the level of the *fla/che* operon in which most flagellum components are encoded (reviewed in 1). *fla/che* contains the gene for the alternative sigma factor σ^D^, which is needed for the transcription of the flagellin gene *hag*, as well as of a number of additional genes. Two promoter sequences drive *fla/che* transcription: P_D3(*fla/che*)_ and P_A(*fla/che*)_ [2]. The σ^A^-dependent P_A(*fla/che*)_ is necessary and sufficient for *fla/che* expression and motility whereas P_D3(*fla/che*)_, which is dependent on σ^D^ for activation, is not sufficient to promote motility and its involvement in a positive feedback effect on *fla/che* expression could not be demonstrated [2,3].

Flagella are necessary for both swimming and swarming motility. Swimming is the typical motility in liquid media, while swarming occurs on semi-solid surfaces. The latter form, described in *B. subtilis* by Kearns and Losick in 2003 [4], requires SwrA to ensure the optimal activation of P_A(*fla/che*)_ transcription which occurs through a still unclear mechanism [5,6]. Additionally, swarming requires surfactin production, which does not occur in laboratory strains because of a mutation in the surfactin biosynthetic pathway [7]. Thus swarming can be analyzed in laboratory strains if two conditions are met: i) the *swrA* allele is in the functional *swrA* ^+^ form; ii) surfactin is added in the medium during the assay [5].

Swimming is boosted by SwrA but it also takes place in its absence, albeit at a reduced rate [8]. The wild-type *swrA* allele is typically found in undomesticated strains; in most laboratory strains, e.g. 168, the *swrA* coding sequence contains a nucleotide insertion that prematurely interrupts its reading frame [5,9]. The inactivating mutation occurs in a mononucleotide repeat sequence and can easily shift back and forth with very high frequency (10^-4^). Thus, the alternation between the functional and non-functional *swrA* alleles is more typical of phase variation mechanisms than point mutations [1,5] and *B. subtilis* cultures are likely to include both *swrA* ^-^ and *swrA* ^+^ cells. 

**Figure 1:**
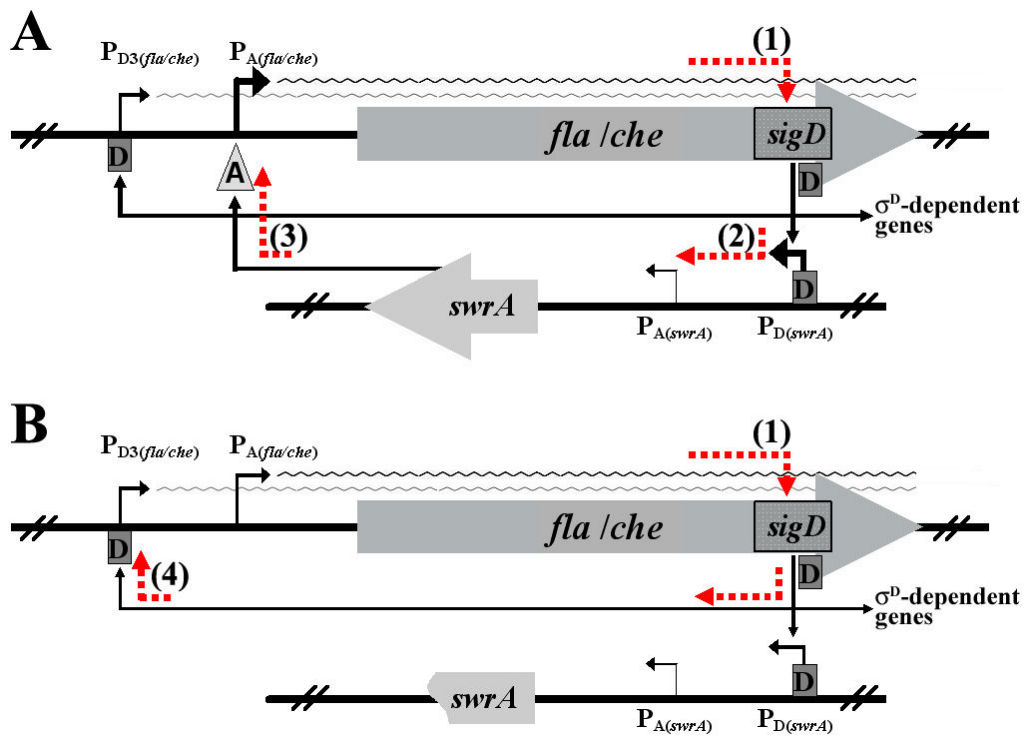
Schematic model for the *fla/che* operon double-autoregulation. The chromosomal regions of *fla/che* and *swrA* are depicted (not to scale); each locus is preceded by its own σ^A^ (P_A_) and σ^D^-dependent (P_D_ or P_D3_) promoter indicated by bent black arrows. *sigD*, the penultimate gene of *fla/che*, is highlighted. Black arrows indicate direct positive effects. Line thickness is proportional to the strength of the effect of each element. A wavy line represents transcripts originating from each *fla/che* promoter. Dashed orange arrows mark the autoregulatory loops that can be predicted, which are identified by numbers in parenthesis. (**A**) In *swrA* ^+^ strains an extremely efficient loop connects SwrA with *fla/che* expression. It starts with *sigD* basal transcription from the P_A(fla/che)_ promoter (1); SigD allows transcription of *swrA* through stimulation of the P_D(swrA)_ promoter (2a). SwrA enhances transcription from P_A(fla/che)_ (3a) closing the circuitry [6, 8]. (**B**) In *swrA* ^-^ strains the closure of the SwrA-based loop is prevented; in these conditions an ancillary and weaker feedback loop takes over. It starts again with *sigD* transcription P_A(fla/che)_ (1); SigD directly activates the weak P_D3(fla/che)_ promoter (4) that transcribes *fla/che* in a positive feedback circuitry. The effect of the weak P_D3(fla/che)_-based loop can be appreciated only in *swrA* ^-^ strains, although it is also active in *swrA* ^+^ strains (see Fig. 6 and text for details).

Transcription of *swrA* is mainly σ^D^-dependent and is positively autoregulated through a circuitry that also sustains *fla/che* expression: SwrA promotes *fla/che* - and thus *sigD* - transcription and σ^D^ transcribes *swrA* (Figure 1A) [8].

Besides the regulatory effect of SwrA, several studies have established that *fla/che* operon expression is also regulated in a complex way by DegS/DegU, a two-component system (TCS) that controls important stationary-phase behaviours in *B. subtilis*. DegU undergoes phosphorylation and dephosphorylation by DegS, which is both a kinase and a phosphatase [10]. It was observed that a non-phosphorylatable DegU mutant causes a non-swarming phenotype in *swrA* ^+^ strains, leading to the conclusion that DegU phosphorylation is required for swarming motility [11,12]. However, employing the *degU3*2(Hy) mutant allele (described below), the repressive effect of DegU~P on motility has been repeatedly observed both in *swrA* ^-^ and *swrA* ^+^ strains [11-16]. It has been proposed that the response regulator DegU can be phosphorylated at different levels allowing it to tune gene expression as a kind of rheostat according to the extent of its phosphorylation. Specifically, at a low level of phosphorylation DegU~P is necessary for swarming; conversely, a highly phosphorylated DegU, as DegU32(Hy), inhibits *fla/che* transcription [10]. 

*degU3*2(Hy) belongs to a group of several mutants that share the pleiotropic “Hy” phenotype which is characterized by the ability to secrete high levels of degradative enzymes, such as proteases and levansucrase, low or no competence, as well as by the lack of flagella [13,17,18]. The “Hy” phenotype is caused by single nucleotide changes in the coding sequence of *degU* or *degS* that perturb the DegS/DegU signal transduction pathway, increasing the stability of phosphorylated DegU in the cell. In *degU3*2(Hy) a H12L amino acid substitution in DegU [18] extends the half-life of the phosphorylated form from 20 to 140 min leading to a higher steady-state level of DegU~P [19]. A very similar phenotype can also be obtained by the over-expression of DegQ or DegR which are small proteins that interfere with the trans-phosphorylation/dephosphorylation cycle between DegS and DegU [13,17,18,20-22]: DegR stabilizes DegU~P [23] and DegQ facilitates phosphate transfer from DegS to DegU [11]. Currently *degU32*(Hy) is the “Hy” mutant most often used in studies on the DegS/DegU TCS and it is considered as an otherwise wild-type more stable DegU~P. 

We have previously hypothesized that DegU can regulate motility cooperatively with SwrA based on evidence that SwrA cannot induce swarming in the absence of a functional *degSU* operon and that deletion of *degS/degU* has no motility phenotype in *swrA* ^-^ strains [8,13]. Furthermore, it was recently shown that SwrA is able to modulate the ability of DegU to activate transcription of *ycdA* [24].

In this work we demonstrate that SwrA forms a complex with DegU~P at P_A(*fla/che*)_ under conditions that correspond to those leading to activation of *fla/che* transcription. We show that DegU~P obtained through the use of DegS200(Hy), a different “Hy” mutant, is not inhibitory on motility when SwrA is functional; conversely, without SwrA, DegU~P produced by *degS200*(Hy) or *degU32*(Hy) is equally repressing P_A(*fla/*che)_. We also show that DegU32(Hy)~P behaves as a constitutive repressor since it does not interact with SwrA on P_A(*fla/che*)_. Overall, our data suggest that DegU phosphorylation, independently of its level, is required for both activation and repression of P_A(*fla/che*)_. Upon phosphorylation, the positive or negative outcome of DegU~P on *fla/che* expression depends on the presence or absence respectively of SwrA. 

Finally, we demonstrate that when the SwrA-based autoregulatory loop is non-functional P_D3(*fla/che*)_ contributes to maintaining *fla/che* expression through an ancillary positive feedback loop (Figure 1B). Thus, P_D3(*fla/che*)_ represents a sort of contingency plan for ensuring a basal level of motility that constitutes a primary survival resource for *B. subtilis*. 

## Materials and Methods

### Bacterial strains and growth conditions

*Bacillus subtilis* strains used in this study with their relevant genotype are listed in Table S1 in Supporting Information. Strains were routinely grown on LB (Luria-Bertani broth: 10 g of tryptone, 5 g of yeast extract, 10 g of NaCl per liter) supplemented with 1.5% agar unless otherwise stated. When required, selective agents were added at the following concentrations: erythromycin 1 or 50 μg ml^-1^, kanamycin 2 μg ml^-1^, spectinomycin 60 μg ml^-1^. The *E. coli* DH5α or BL21 and BL21(DE3) strains were grown at 37°C in LB with 100 μg ml^-1^ ampicillin and 20 μg ml^-1^ chloramphenicol when required. 

### Strain construction

**Figure 2:**
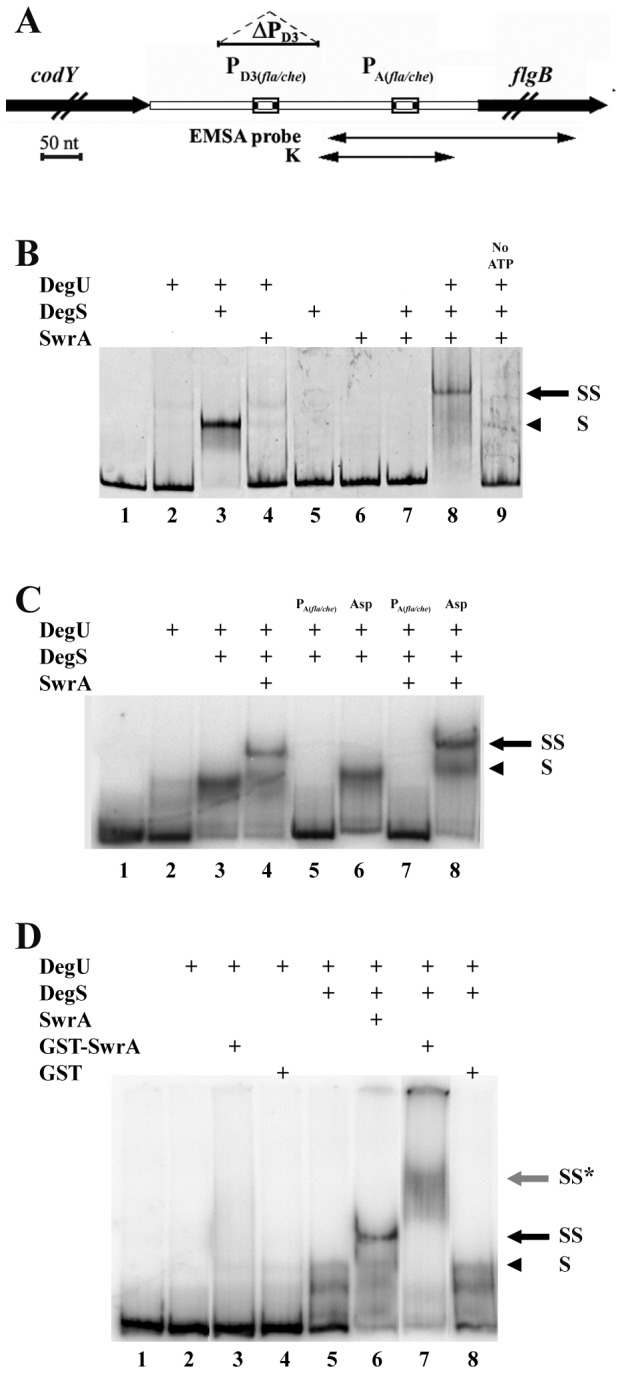
SwrA is bound to P_A(fla/che)_ through DegU~P. (**A**) The region of the *fla/che* promoter, including the σ^A^ and σ^D^-dependent promoters, is drawn to scale. The coding sequences of the preceding gene (codY) and of the first gene of *fla/che* (flgB) are in black. The span of the P_A(fla/che)_ probe, of the K probe used in Figure S1 and of the markerless P_D3_ deletion are indicated. The bar corresponds to 50 nt. (**B**) EMSA with fluorescently labeled P_A(fla/che)_ probe. DegU, DegS and SwrA (listed on the left above the gel) were added to reactions labeled with a + above the lane. In each 10 μL-reaction proteins had the following concentrations: 0.35 μM for DegU; 0.4 μM for DegS; 1 μM for SwrA. ATP was omitted from the reaction in lane 9. On the right of the gel, the arrowhead labeled with S points to the probe shifted by DegU~P (lane 3); the SS arrow marks the super-shifted complex formed upon SwrA addition (lane 8). (**C**) Competition reactions with an excess (20 ng) of unlabeled P_A(fla/che)_ DNA or with the same amount of unlabeled non-specific (N-S) DNA are indicated above the gel. The EMSA probe was radioactively labeled. In each reaction, the proteins had following concentration: 0.2 μM DegU, 0.1 μM DegS and 0.2 μM SwrA. (**D**) A GST-tag fused to SwrA reduces the migration of the SS complex. Radioactively labeled P_A(fla/che)_ was incubated with DegU (0.1 μM), DegS (0.1 μM), and either SwrA (lane 6), GST-SwrA (lane 7) or GST (lane 8), each at 1.5 μM. The grey arrow labeled with SS* points at the retarded band obtained with GTS-SwrA (lane 7).

Plasmids and oligonucleotides used in this work are listed in Tables S2 and S3 respectively, in Supporting Information. The markerless P_D3(*fla/che*)_ deletion, that removes 118 bp from the *fla/che* upstream region (between positions -302 and -184 from the *flgB* translation start site) (Figure 2A), was generated with pMADΔPD3. For its construction two separate PCR fragments containing the two flanking regions of P_D3(*fla/che*)_ were amplified from genomic DNA using the primer pairs CodYF and 8465 (upstream region) and 8601 and nRflgBN (downstream region). The two fragments were blunt-end ligated and re-amplified with the external primers CodYF and nRflgBN. The restricted PCR product was ligated into the *Eco*RI and *Nco*I site of the pMAD vector [25]; the construct was verified by sequencing. pMADΔPD3 was used to transform PB5392, PB5394 and PB5396 following the procedure described [25], in order to obtain strains PB5455, PB5458 and PB5466 respectively.

The markerless *dhsA6* mutation in P_A(*fla/che*)_ in strain PB5452 was generated by transformation of PB5370 with pMADdhs (Table S2), as already reported [26]. 

### Motility assays

Cells, taken from a fresh culture grown on LB plates, were seeded at the centre of motility plates (85 mm) with a toothpick, taking special care not to disturb the surface. Swimming motility was evaluated on freshly prepared semiliquid LB plates containing 0.2% of agar. Pictures were taken after 13 hours of incubation at 30°C. Swarming motility was evaluated on LB plates containing 0.7% of agar and surfactin, as already reported [8]. All motility experiments were independently repeated more than thirty times each. For swimming expansion assays, 150 mm-swimming plates were incubated at 37°C and the extension of swimming halos was recorded every hour. Assays were independently repeated three times and processed with Microsoft Excel for plotting and standard deviation calculation.

### Western blot analysis

Cells were inoculated in LB liquid medium with a starting optical density at 600 nm (OD_600_) of 0.2. One hour after the transition phase 4 x 10^8^ cells were collected by centrifugation and pellets were dissolved in 100 μl of SDS-PAGE sample buffer 2X (125 mM Tris-HCl pH 6.8, 4% SDS, 50% glycerol, 10% β-mercaptoethanol, 0,02% bromophenol blue), sonicated twice for 12 sec and boiled for 5 min. One tenth of each sample was separated on a 12% SDS-PAGE gel, then transferred to a nitrocellulose filter. Homogeneity of cell lysates was checked by Ponceau staining. The blot was probed with a rabbit anti-flagellin polyclonal antibody [27], detected with a HRP-conjugated anti-rabbit secondary antibody.

### Recombinant protein production and purification

For DegU and DegU32(Hy) production, competent *E. coli* BL21(DE3) cells were transformed with pET16Uwt or pET16Uhy plasmids (Table S2), grown at 37°C and induced with 1 mM of IPTG for 3 hours at 28°C. Cells were collected by centrifugation, resuspended in 0.02 volumes of buffer A (20 mM Tris-HCl pH 8, 300 mM NaCl, 1% Triton X-100, 0.5 mM PMSF, 5 μg ml^-1^ DNaseI, 20 mM imidazole) and sonicated. Cell lysate was centrifuged at 10,000 g at 4°C for 15 minutes to remove cellular debris; the supernatant was incubated with Co-IDA agarose beads (Biontex), previously equilibrated in buffer B (20 mM Tris-HCl pH 8, 300 mM NaCl, 20 mM imidazole), at 4°C for 1 hour. After extensive washing with buffer B, proteins were eluted with 500 mM imidazole in buffer B; protein-containing fractions were pooled and dialyzed against buffer C (20 mM Tris-HCl pH 8, 1 mM EDTA). The dialyzed proteins were loaded on a 5 ml DEAE column (GE Healthcare) to remove any contaminant DNA and eluted with a discontinuous NaCl gradient; after SDS-PAGE analysis, selected fractions were dialyzed against buffer D (20 mM Tris-HCl pH 8, 200 mM NaCl, 1 mM EDTA, 50% [v/v] glycerol) and stored at -80°C in aliquots.

*degS* and *degS200*(Hy) genes were amplified from PB5249 or PB5390 (Table S1), respectively, with primers DegSFcacc and DegSRblunt. Products were cloned into the directional pET101/D-TOPO plasmid (Invitrogen) and plasmids were sequenced. The resulting pETOPO-DegS or pETOPO-DegSHy together with pGro7 (Takara Bio) were used to transform *E. coli* BL21(DE3) according to the manufacturer’s protocol. DegS and DegS200Hy were purified according to the same protocol used for DegU and DegU32Hy, omitting the DEAE step. GST-SwrA and SwrA purification has already been described [27]. All proteins were dialyzed against buffer D, which was also used to normalize protein volumes in EMSA. Protein purity was assessed by SDS-PAGE and concentration was calculated through absorbance at 280 nm and with a Bradford Protein Assay (Bio-Rad). 

### EMSA

EMSA were performed using two different labeling and detection procedures. The P_A(fla/che)_ probe (Figure 2A) was amplified from PB5249 DNA either with primers cs8610 and nRflgBN (285 bp) and end-labeled with [γ-^32^P]-ATP using T4 polynucleotide kinase, or with 6-FAM-fluorescent-labeled primers 8601 and nRFlgB (299 bp). A shorter K probe (156 bp, Figure 2A), amplified with 6-FAM-fluorescent-labeled primer 8601 and a non-labeled primer 8756, was also used in EMSA (Figure S1). Probes were purified by silica microcolumns. Binding reactions (10 μl) contained: 12 fmoles of labeled dsDNA, 0.1 μg of sonicated salmon sperm DNA, 0.1 mM ATP, binding buffer BB (50 mM Tris-HCl pH 7.6, 10 mM MgCl_2_, 1 mM DTT, 25 mM KCl, 0.1 mg ml^-1^ BSA) and a total volume of 3 μl of the required proteins in different final concentrations, as specified in each figure legend; volumes were normalized to 3μl using buffer D. Reaction mixtures were incubated for 10 min at room temperature (26°C), in the dark for FAM-labeled probes, and were then resolved on a native 5% polyacrylamide gel (6.7 mM Tris-HCl pH 7.6, 3.3 mM Na-acetate, 1 mM EDTA, 5% acrylamide, 2.5% glycerol). The gel was pre-run at 120 V at 4°C, in a low ionic strength TAE buffer (6.7 mM Tris-HCl pH 7.6, 3.3 mM Na-acetate, 1 mM EDTA) for 1 hour; samples were separated at 190 V at 4°C for 3 hours. Gels were either dried for radioactive signal acquisition with a Cyclone Plus Phosphor Imager system (Perkin Elmer), or directly analyzed with a Typhoon scanner (GE Healthcare) for FAM-labeled substrates. The unlabeled P_A(*fla/che*)_ was used as a specific competitor in Figure 2C, while an internal region of *degS*, amplified with primers R3 and S3 as in [11], was used as a non-specific competitor.

## Results

### SwrA binds to the fla/che promoter together with DegU~P

Previous work has demonstrated a positive effect of the functional SwrA protein on *fla/che* transcription [6] but the method of operation of SwrA has remained unclear. SwrA does not contain any known DNA binding domain, therefore its effect on *fla*_*/*_ *che* is unlikely to be due to direct binding to the P_A(fla/che)_ promoter. However, an indirect interaction with P_A(*fla/che*)_ through a bridging factor has never been explored. The discovery that DegU binds to P_A(*fla/che*)_ [11,16] as well as the evidences suggesting a cooperative effect between DegU and SwrA [8,24], prompted us to investigate the possible interaction of the two proteins at the P_A(fla/che)_ locus. Initially, pull-down assays were attempted using SwrA, DegU and DegS proteins in several different conditions and prey/bait combinations but the observed strength of interaction was not satisfactory. Indeed, difficulties to assess an interaction between SwrA and DegU have been previously noted [24]. Two possible reasons account for this result: i) the interaction between these two proteins, which is mediated by a transient phosphorylation event (described below), is intrinsically unstable *in vitro*; ii) DegU, DegS and SwrA bind to Glutathione and Nickel-charged resins aspecifically, in all the conditions tested, causing residual signals in the negative control reactions. As an alternative, EMSA are reported to be more sensitive than other types of assay for the detection of unstable interactions as the gel matrix intervenes to stabilize labile complexes through a “caging” effect [28]. Therefore, SwrA, DegU and DegS were challenged with a PCR probe (ca 300 bp) spanning the P_A(fla/che)_ region (Figure 2A) in EMSA. 

**Figure 4:**
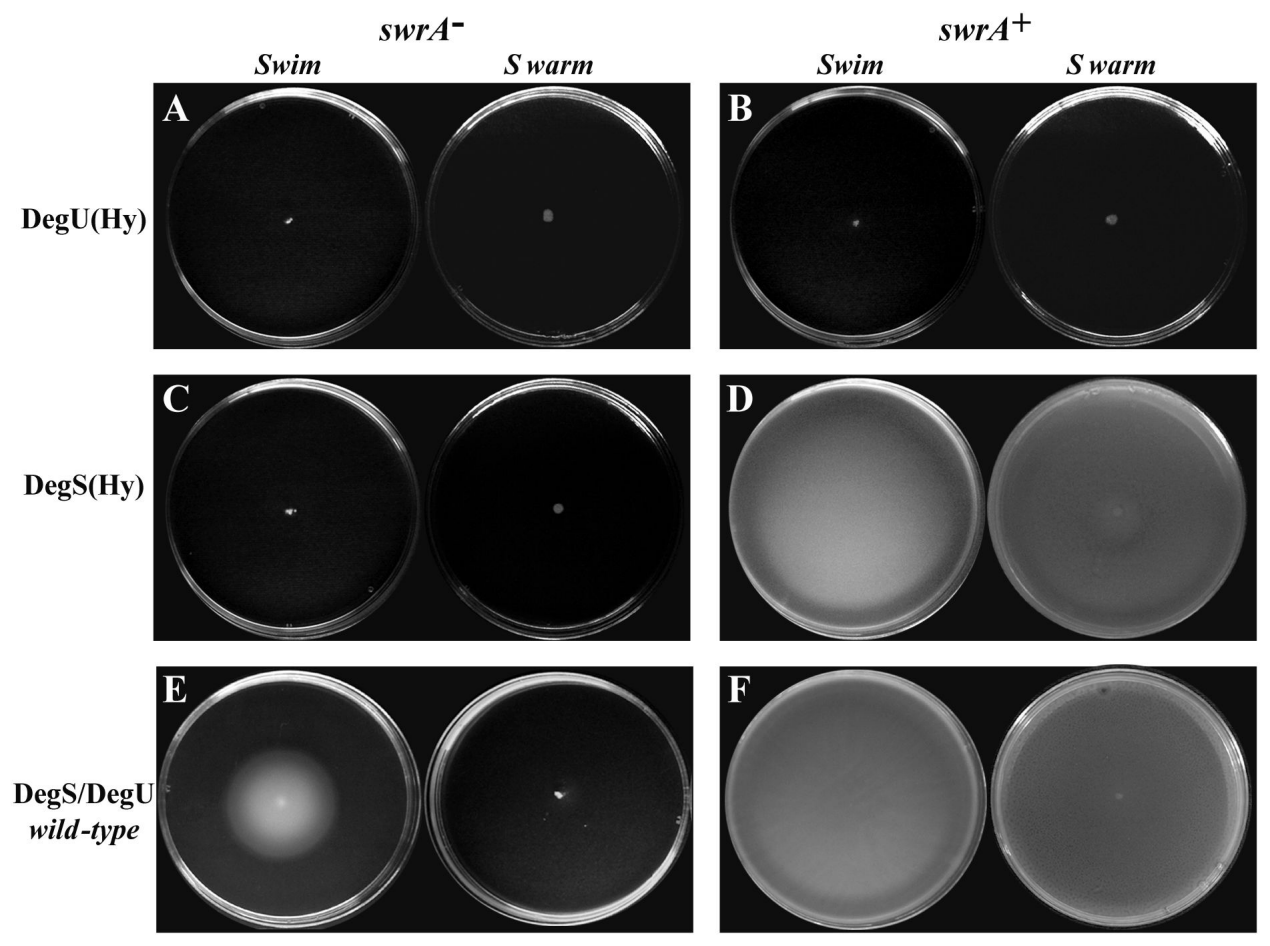
DegU32(Hy) and DegS200(Hy) behave differently in EMSA. Fluorescently labeled P_A(fla/che)_ was challenged with the mutant DegU32(Hy) or DegS200(Hy) proteins in EMSA. DegS200(Hy) was used to phosphorylated wild-type DegU in lanes 10 and 11. DegU32(Hy) was phosphorylated by wild-type DegS in lanes 5 and 6. Protein concentrations were the following: 0.2 μM for DegS; 0.4 μM for DegS200(Hy); 0.36 μM for DegU and 1 μM for SwrA. Different concentrations of DegU32(Hy) were used: 0.3 μM in lane 2; 0.6 μM in lanes 3, 5 and 6; 1.8 μM in lane 4. Control reactions with wild-type proteins are shown in lanes 7, 8, 9. An asterisk marks the particular pattern of bands, bracketed on the left-hand side of the gel, produced by DegU32(Hy) and DegU32(Hy)**~**P. Other symbols are the same as in Figure 2B.

We observed that DegU showed higher affinity for P_A(*fla/che*)_ when phosphorylated by DegS kinase (Figure 2B) as previously reported [11], and formed a complex with DNA whose mobility is marked with an “S” in Figure 2. ATP was required for the interaction (data not shown). However, when SwrA was added to the reaction containing DegS and DegU the probe was super-shifted in a complex (SS complex in Figure 2) with a lower mobility than the S complex (DegU~P:DNA). The SS complex was not assembled when ATP was omitted from the reaction, thus indicating active DegU phosphorylation was required. SwrA did not bind the DNA probe, either alone or in combination with DegS or with unphosphorylated DegU. To verify the specificity of both S and SS complexes an excess of either the unlabelled P_A(*fla/che*)_ probe or an unrelated DNA fragment was added to the reactions. Whereas the unlabeled P_A(*fla/che*)_ was able to outcompete the labeled probe, the non-specific DNA fragment could not (Figure 2C) thus confirming that both complexes were specifically occurring on P_A(*fla/che*)_. Both the S and SS complexes were also obtained with a shorter probe, K, spanning a narrower region (156 bp) around P_A(*fla/che*)_ (Figure 2A and Figure S1). The robustness of S and SS complexes was corroborated by their reproducibility over a wide range of concentrations of the three proteins used in the reactions, as indicated in the legends of Figures 2, 4, S1 and S2.

Next we sought to determine whether the slower migration of the SS complex was dependent on an SwrA-induced conformational change of DegU~P:DNA or rather on the association of SwrA with the S complex. To discriminate between these two possibilities SwrA (14 kDa) was substituted in EMSA by a higher molecular mass GST-SwrA fusion protein (41 kDa). No differences with the untagged SwrA were expected in the case that SwrA had an indirect effect on mobility of the complexes, whereas a further decrease in migration of the SS complex was anticipated in the case that SwrA was integral to the DNA-bound complex. As shown in Figure 2D, the sole GST moiety used as a control did not interfere with the assay. GST-SwrA behaved as the untagged SwrA: it did not interact with DNA alone (not shown) or in combination with non-phosphorylated DegU, whereas it was able to super-shift the probe in the presence of DegU~P (SS* in Figure 2C, lane 7). In this case, however, the mobility of the complex was indeed slower than the SS complex obtained with untagged SwrA, demonstrating that SwrA was physically associated with the DNA-bound complex. Moreover, in reactions in which both DegU and DegS concentrations were limiting we observed a substantial increase in the efficiency of DNA binding in the presence of SwrA (Figure S2).

Thus, SwrA modifies the mobility of DegU~P:P_A(*fla/che*)_ by taking part in the complex and increasing its affinity for DNA. DegU phosphorylation is required for such an interaction.

### The role of phosphorylation on DegU and the nature of DegU32(Hy)

**Figure 3:**
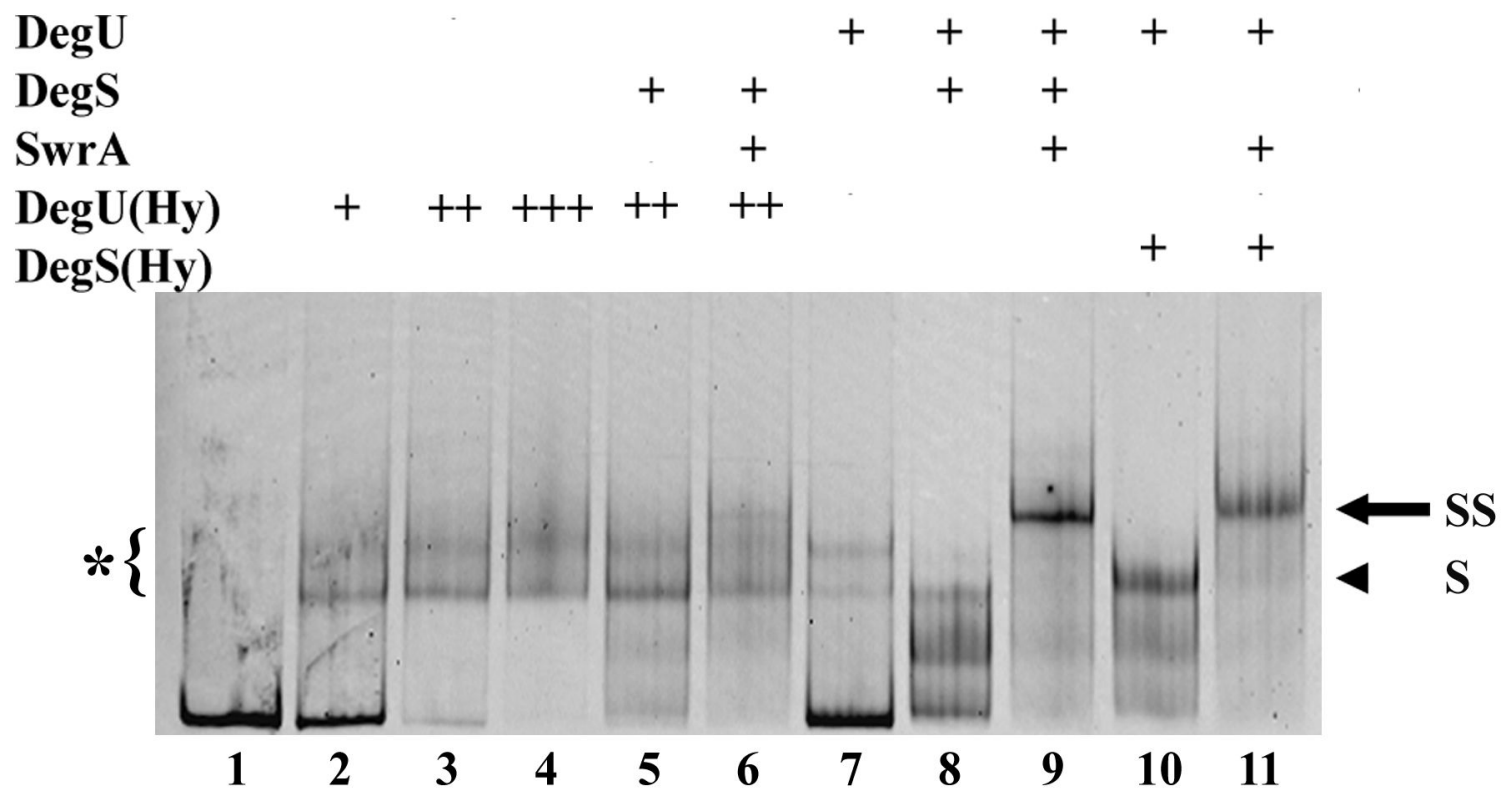
Motility is not repressed by high levels of DegU~P in the presence of SwrA. Swimming (on the left of each panel) and swarming (on the right) motility assays for strains differing in the status of the *swrA* allele (indicated on top of each panel) and of the *degS/degU* alleles (indicated on the left). Each strain was inoculated at the center of motility plates and images were taken after 13 hr of incubation. Strains are: PB5384 (A) and PB5383 (B) for *degU32*(Hy); PB5391 (C) and PB5390 (D) for *degS200*(Hy); PB5370 (E) and PB5249 (F) for wild-type *degS/degU*.

Our results indicate that DegU phosphorylation is necessary for the interaction with the swarming factor SwrA, in line with previous work that highlighted the necessity of a phosphorylatable DegU for swarming [11,12]. However, it has also been repeatedly observed that high levels of DegU~P, as obtained with the *degU32*(Hy) allele, cause a non-motile phenotype independent of the status of the *swrA* gene [11-16]. In order to investigate the relevance of the extent of phosphorylation of the transcription factor DegU we analyzed motility under conditions in which a wild-type DegU is highly phosphorylated. For this purpose we used the *degS200*(Hy) mutant allele which causes a pleiotropic phenotype whose characteristics and severity are indistinguishable from that of *degU32*(Hy) [13,17]. *degS200*(Hy) leads to a G218E substitution in DegS that impairs its dephosphorylation activity leading to accumulation of a wild-type DegU~P [29]. When strains were transformed with the *degS200*(Hy) allele swimming and swarming motility (on surfactin-supplemented plates) greatly differed between the isogenic *swrA* ^*+*^ and *swrA* ^-^ backgrounds (Figure 3). In the *swrA* ^-^ *degS200*(Hy) strain swimming was repressed compared to the wild-type *degS/degU* sibling, in line with previous reports and with what occurs with *degU32*(Hy). Conversely, in the *swrA* ^*+*^ background the accumulation of the DegU~P form mediated by *degS200*(Hy) did not preclude robust swimming and swarming motility. 

These findings indicate that the higher extent of DegU phosphorylation caused by impairment of the phosphatase activity of DegS does not hinder motility when SwrA is present, but is indeed repressive when SwrA is absent. Therefore, the level of phosphorylation attained by DegU is not the cause of motility inhibition *per se*. Indeed, wild *B. subtilis* strains carry the *degQ36*(Hy) allele that increases the phosphorylation rate of DegU but are nonetheless swarming proficient [11].

To elucidate the reason for the different behavior of *degU32*(Hy) and *degS200*(Hy) alleles, the corresponding mutant proteins were purified and challenged with the P_A(fla/che)_ probe in EMSA. As shown in Figure 4, DegS200(Hy) behaved exactly as its wild-type counterpart: it was able to phosphorylate wild-type DegU and induced the formation of the SS complexes in the presence of SwrA. Conversely, DegU32(Hy) bound DNA efficiently, even at low concentration and in the absence of DegS, causing a distinctive band-shift pattern (indicated by an asterisk in Figure 4). This pattern was not substantially modified by the addition of DegS to the reaction and was not efficiently converted into the SS complex upon addition of both DegS and SwrA. Since the mutant DegU32(Hy) protein can be trans-phosphorylated and dephosphorylated by DegS *in vivo* [13] and *in vitro* [19], the absence of the SS complex was not due to a lack of phosphorylation. Thus, the most likely explanation is that the H12L amino acid substitution in DegU32(Hy) not only causes a slow dephosphorylation rate, as already assessed [19], but also impairs its interaction with SwrA at P_A(*fla/che*)_, preventing the assembly of the SS complex. In agreement with this hypothesis, the H12L mutation is localized in the N-terminal domain of DegU, which is the domain that interacts with SwrA in Yeast-2-Hybrid assays [24].

The results obtained *in vivo* and *in vitro* with DegU32(Hy) and DegS200(Hy) demonstrate that DegU phosphorylation, even at high levels, is not necessarily linked to motility repression; rather, that the ability to interact with SwrA is crucial for swarming and swimming. Indeed, in the absence of SwrA repression of *fla/che* transcription occurs with both DegU32(Hy) and DegS200(Hy), indicating that DegU~P by itself acts as a P_A(fla/che)_ repressor. The full motility of the *degS200*(*Hy*) *swrA* ^*+*^ strain *in vivo* coupled with the ability of wild-type DegU~P [but not DegU32(Hy)] to interact with SwrA at P_A(*fla/che*)_ *in vitro* suggests that the SS complex is the positive stimulator of *fla/che* transcription.

**Figure 5:**
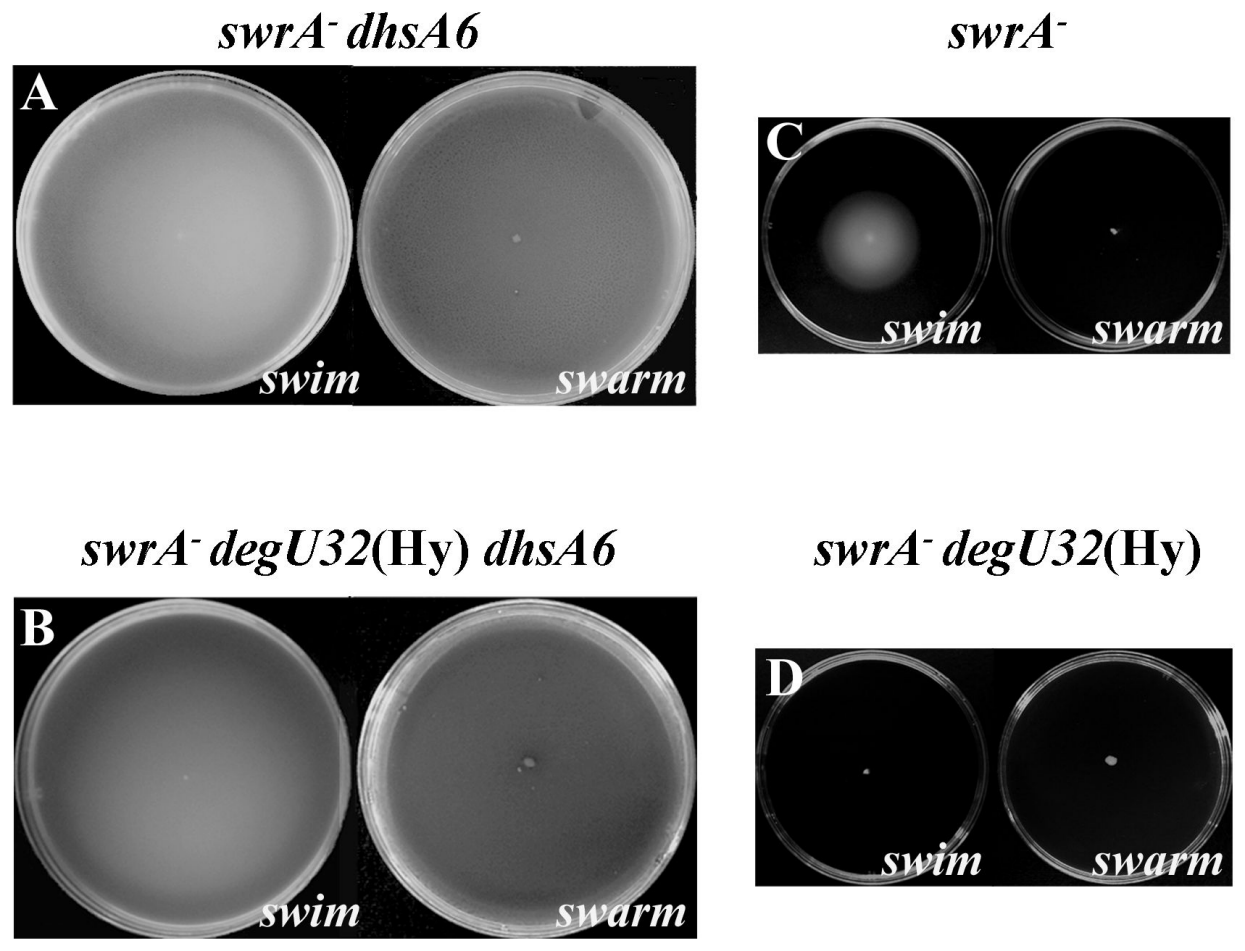
Motility defects caused by the *swrA* ^-^ and *degU32*(Hy) alleles are suppressed by an optimized P_A(fla/che)_. In the A and B panels swimming (left) and swarming (right) performances of *swrA* ^-^ or *swrA* ^-^ *degU32*(Hy) strains containing the *dhsA6* mutation is respectively shown. The single nucleotide *dhsA6* mutation in P_A(fla/che)_ improves similarity to the σ^A^ consensus sequence [16] and thus the predicted promoter strength. Motility of the parental *swrA* ^-^ and *swrA* ^-^ *degU32*(Hy) strains is shown in C and D respectively. Relevant genotypes are indicated on top of each panel. Strains used to generate the images are: PB5452 (A); PB5447 (B); PB5370 (C); PB5384 (D).

To test whether such a trigger is sufficient to relieve DegU32(Hy)-mediated swarming inhibition we sought to analyze motility in a *degU32*(Hy) *swrA* ^-^ background in which an optimized P_A(*fla/che*)_, with an increased similarity to the σ^A^ consensus sequence, was introduced. To this end we used the *dhsA*6 (*degUHy* suppressor) single-nucleotide mutation in the -35 box of P_A(*fla/che*)_ that has been shown to suppress the motility-null phenotype caused by *degU32*(Hy) [16]. Swarming was not assessed in the original *dhsA*6 mutant since SwrA had not been identified yet. As shown in Figure 5, both swimming and swarming were fully recovered in the strain carrying the *dhsA*6-optimized P_A(*fla/che*)_ promoter, although the constitutive *degU32*(Hy) mutation was still active and the functional *swrA* allele was absent. This result confirms that the enhancement of *fla/che* transcription is sufficient for swarming.

### The role of P_D3(fla/che)_

After having investigated the mechanisms which promote *fla/che* transcription from P_A(*fla/che*)_, the role of the secondary σ^D^-dependent *fla/che* promoter was evaluated. Although a feedback effect of P_D3(*fla/che*)_ in maintaining *fla/che* expression is postulated (Figure 1B), previous studies have failed to detect it [3]. We have already shown the existence of a powerful SwrA-dependent autoregulatory circuitry (Figure 1A) [8]. We reasoned that the existence of such an efficient loop might mask the effect of P_D3(*fla/che*)_ and would therefore account for the lack of evidence reported in the past. 

**Figure 6:**
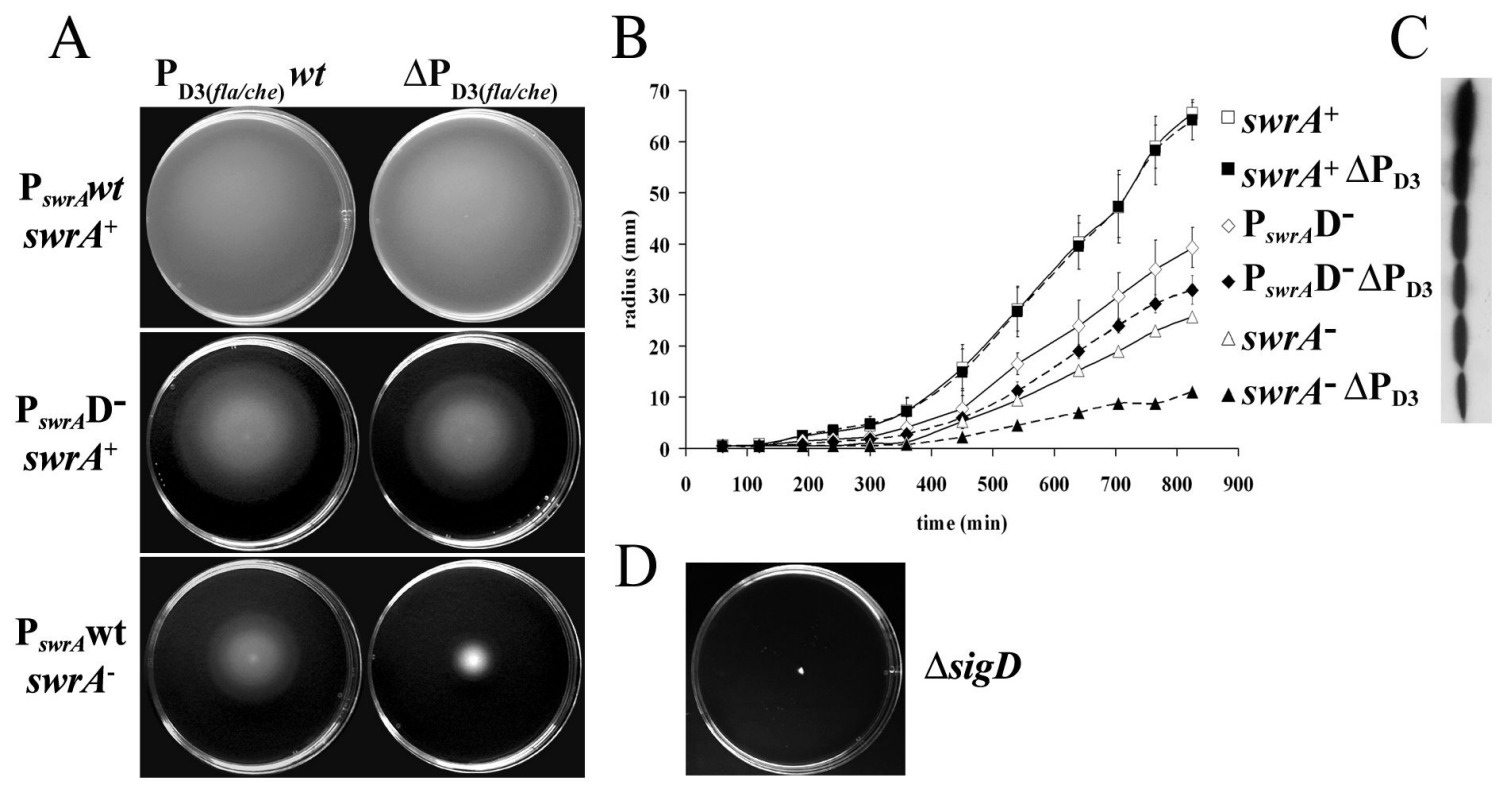
The contribution of P_D3(fla/che)_ to motility is masked by SwrA. The effect of a P_D3(fla/che)_ deletion on motility was evaluated in strains differing in the status of the *swrA* allele or of the σ^D^-dependent *swrA* promoter. The isogenic strains used are PB5392 [*swrA* ^+^ P_D3(fla/che)_wt], PB5455 [*swrA* ^+^ ΔP_D3(fla/che)_], PB5394 [P_*swrA*_D^-^-*swrA* ^+^ P_D3(fla/che)_wt], PB5458 [P_*swrA*_D^-^-*swrA* ^+^ ΔP_D3(fla/che)_], PB5396 [*swrA* ^-^ P_D3(fla/che)_wt] and PB5466 [*swrA* ^-^ ΔP_D3(fla/che)_]. (**A**) Swimming assays. Genotypes are indicated on the left and on top of each panel. (**B**) Swimming expansion measurements. Relevant genotypes are specified on the right. Results shown are an average of three independent experiments; error bars correspond to standard deviation. (**C**) Western blot of flagellin collected at T_1_ from liquid cultures of the strains used in A and B, vertically aligned to the bullets identifying the strains in B. (**D**) Swimming assay for a Δ*sigD* *swrA* ^+^ strain (PB5427) shown as reference for non-motile behavior.

To understand the role of P_D3(*fla/che*)_ we used strains in which the SwrA-based loop was impaired, either through a mutation in the σ^D^-dependent SwrA promoter P_*swrA*_D^-^ in front of the *swrA* ^+^ allele to interrupt the circuitry at the level of *swrA* transcription (Figure 1A), or through the frameshifting mutation in *swrA* that is found in laboratory strains [5]. The effect on swimming motility of the P_*swrA*_D^-^ mutation is mild because it is partially relieved by the presence of the P_*swrA*_A promoter; in fact, this strain is swarming proficient because the P_*swrA*_A promoter becomes fully active on solid surfaces [8]. Conversely, the frameshifting mutation causes a more severe phenotype in swimming and prevents swarming [5]. A markerless 118-nucleotide deletion of P_D3(*fla/che*)_ (ΔP_D3_ in Figure 2A) was created in the two strains as well as in a *swrA* ^+^ control. We decided to analyzed swimming in order to measure motility performances even in the *swrA* ^*-*^ backgrounds in which swarming is precluded. As shown in Figure 6A, swimming was not affected by the ΔP_D3_ mutation in the control *swrA* ^+^ strain. However, deletion of P_D3_ caused a progressively more severe phenotype in the *swrA* mutant backgrounds, exacerbating the effect of mutations in *swrA*. A time course of swimming expansion allowed a better appreciation of the increasingly greater differences between each ΔP_D3_ strain and its P_D3_-wild-type counterpart (Figure 6B). A parallel decrease in the amount of flagellin produced by these strains was also observed during growth in shaking cultures (Figure 6C). 

These data demonstrate that P_D3(*fla/che*)_ is indeed involved in positive feedback regulation of *fla/che* transcription, thereby increasing flagellin production and swimming speed, but its effect can be appreciated only in the absence of SwrA. The severe motility phenotype of our *swrA* ^-^ ΔP_D3_ mutant strongly corroborates the importance of P_D3_ in motility autoregulation. Thus, P_D3(*fla/che*)_ is redundant in *swrA* ^+^ strains but it ensures basal motility in case the *swrA* allele switches to the non-functional status. 

We surmise that the discrepancy between our results and those of previous investigations [3] lies in the fact that those experiments were performed in *swrA* ^-^ laboratory strains containing spontaneous *swrA* ^+^ revertants; thus, the heterogeneous *swrA* background would have masked the contribution of P_D3(*fla/che*)_. Indeed, several *swrA* ^+^ revertants arose during motility evaluation of *swrA* ^-^ strains due to the very high frequency of forward and reverse mutations that occur in the “slippery” polyadenine tract of *swrA* [5], and scrupulous analysis of the status of the *swrA* allele was performed immediately before and after each experiment (see also 30). 

## Discussion

Results contributing to a clearer picture of the regulatory signals mediating *fla*/*che* transcription have been presented. We have demonstrated that SwrA interacts with phosphorylated DegU and forms a complex on the *fla/che* promoter. This complex presumably represents the SwrA-mediated *fla/che* positive trigger that is mandatory for activating swarming motility. Thus, although flagella are assembled and support basal swimming even the absence of SwrA and DegU [8,13], phosphorylation of DegU is necessary for swarming motility as earlier demonstrated [11,12,14], its phosphorylation being necessary for the interaction with SwrA, although it is repressive in the absence of SwrA as previously observed [13,15,16]. The molecular function of SwrA is to act as a modifier of DegU~P, converting it from a repressor to a booster of *fla/che* expression. Interestingly, the availability of SwrA is modulated through a phase variation ON/OFF mechanism, thereby ensuring diversification of motility performances upon expansion of a clonal population.

Our findings define a situation in which DegU is a dual regulator: upon phosphorylation, which possibly occurs through inhibition of flagella rotation [31], it can act as a repressor or a stimulator of *fla/che* transcription; the decision is made through an association with the regulator SwrA. Therefore SwrA, together with DegQ and DegR which modulate the phosphorylation/dephosphorylation rate of DegU, represent distinct auxiliary factors that intervene in the fine tuning of the DegS/DegU response, possibly merging different signals directed to this crucial TCS. The action of auxiliary factors is not unusual in TCS signaling pathways, as recently reviewed [32]. 

Presumably, SwrA also has a more general effect in addition to its specific effect on P_A(*fla/che*)_ illustrated here, and might contribute to modulating the expression of other genes belonging to the DegU~P regulon. Indeed, besides *ycdA* [24], preliminary data indicate that in a wild-type *degS/degU* background the production of extracellular proteases, that is normally triggered by “Hy” mutations [13,17,19], can be modulated by SwrA (Figure S3). Unfortunately, most of the studies on the DegU regulon have been carried out using the *degU*32(Hy) allele which we have demonstrated to be a mutant protein impaired in its interaction with SwrA; thus, the effects of SwrA on DegU-regulated genes have probably been missed. It would be worthwhile analyzing DegU~P-dependent processes in *swrA* ^+^ backgrounds by using the *degS200*(Hy) mutant allele.

We have also shown that P_D3(*fla/che*)_ allows the establishment of a positive autoregulatory loop that is used in maintaining *fla/che* transcription when SwrA is absent (Figure 1B). The importance of this feedback loop is that it could contribute to explain the bistable switch that governs motility in *B. subtilis*. In fact, motility is virtually null in the *swrA* ^-^ ΔP_D3_ (Figure 6); the few residual motile cells could be due to the limited amount of functional SwrA produced through transcriptional slippage, as recently proposed by Gordon and collaborators [33]. These authors have elegantly shown that in heritable ON/OFF epigenetic switches whose regulator is inactivated by a slippery A_9_ tract in its coding sequence (such as *swrA*) transcription errors can generate wild-type mRNA, and thereby enough active regulator, to promote epigenetic ON-switching. Further work is required to gather data to verify this hypothesis.

Finally, it is remarkable that although a single nucleotide change might have turned P_A(*fla/che*)_ into a self-sufficient promoter, this promoter has been maintained as intrinsically suboptimal: the possibility of regulating energetically-demanding flagellar motility over a wider dynamic range probably has an essential adaptive value. 

## Supporting Information

Figure S1 **The S and SS complexes assemble also on a shorter probe K.** EMSA were performed as described in Material and Methods with FAM-labeled probes. The final concentration (μM) of the proteins present in each reaction is indicated on top of the figure. In the first seven lanes reactions were performed with the standard P_A(fla/che)_ probe used in Figures 2 and 4 of the manuscript; in the following seven lanes the shorter 156bp-probe K was used (depicted in Figure 2A). Probe K was amplified with FAM-labeled primer pair 8601 and 8756 (Table S3).
(TIF)

Figure S2 **SwrA increases the DNA binding affinity of DegU~P.** EMSA were performed with the standard radioactively-labeled P_A(fla/che)_ as described in Material and Methods. DegU, DegS and SwrA were used in limiting concentrations as specified below each lane (final μM concentration in each reactions). The increase in affinity is evident by comparing lanes 6 and 10.
(TIF)

Figure S3 **Extracellular protease secretion is enhanced by SwrA.** Extracellular protease production was evaluated as previously described [12] by means of skim milk plates. DegU32(Hy) induces proteases secretion in either *swrA* ^+^ and *swrA* ^-^ isogenic strains as already published [13,17,19]. In a wild-type *degS/degU* background, though, also the SwrA- containing strain produces a higher level of proteases compared to the isogenic *swrA* ^-^ strain. Strains used to generate this image are: PB5249, PB5370, PB5383, PB5384 whose genotypes are listed in Table S1. The control strain carrying the *swrA* deletion (PB5334) has been previously described [27].
(TIF)

Table S1 **Strains used in this study.**
(DOCX)

Table S2 **Plasmids used in this study.**
(DOCX)

Table S3 **Primers used in this study.**
(DOCX)

## References

[1] Patrick JE, Kearns DB (2012) Swarming motility and the control of master regulators of flagellar biosynthesis. Mol Microbiol 83: 14-23. doi:10.1111/j.1365-2958.2011.07917.x. PubMed: 22092493. 22092493 PMC3245337

[2] Estacio W, Santa Anna-Arriola S, Adedipe M, Márquez-Magaña LM (1998) Dual promoters are responsible for transcription initiation of the *fla*/*che* operon in *Bacillus**subtilis*. J Bacteriol 180: 3548-3555 10.1128/jb.180.14.3548-3555.1998 PMC107321 9657996

[3] West JT, Estacio W, Márquez-Magaña L (2000) Relative roles of the *fla/che* P_A_, P_D-3_, and P_sigD_ promoters in regulating motility and *sigD* expression in *Bacillus* *subtilis* . J Bacteriol 182: 4841-4848. doi:10.1128/JB.182.17.4841-4848.2000. PubMed: 10940026. 10940026 PMC111362

[4] Kearns DB, Losick R (2003) Swarming motility in undomesticated *Bacillus* *subtilis* . Mol Microbiol 49: 581-590. PubMed: 12864845. 12864845 10.1046/j.1365-2958.2003.03584.x

[5] Kearns DB, Chu F, Rudner R, Losick R (2004) Genes governing swarming in *Bacillus* *subtilis* and evidence for a phase variation mechanism controlling surface motility. Mol Microbiol 52: 357-369. doi:10.1111/j.1365-2958.2004.03996.x. PubMed: 15066026. 15066026

[6] Kearns DB, Losick R (2005) Cell population heterogeneity during growth of *Bacillus* *subtilis* . Genes Dev 19: 3083-3094. doi:10.1101/gad.1373905. PubMed: 16357223. 16357223 PMC1315410

[7] Nakano MM, Corbell N, Besson J, Zuber P (1992) Isolation and characterization of *sfp*: a gene that functions in the production of the lipopeptide biosurfactant, surfactin, in *Bacillus* *subtilis* . Mol Gen Genet 232: 313-321. PubMed: 1557038. 1557038 10.1007/BF00280011

[8] Calvio C, Osera C, Amati G, Galizzi A (2008) Autoregulation of *swrAA* and motility in *Bacillus* *subtilis* . J Bacteriol 190: 5720-5728. doi:10.1128/JB.00455-08. PubMed: 18567663. 18567663 PMC2519373

[9] Zeigler DR, Prágai Z, Rodriguez S, Chevreux B, Muffler A et al. (2008) The origins of 168, W23, and other *Bacillus* *subtilis* legacy strains. J Bacteriol 190: 6983-6995. doi:10.1128/JB.00722-08. PubMed: 18723616. 18723616 PMC2580678

[10] Murray EJ, Kiley TB, Stanley-Wall NR (2009) A pivotal role for the response regulator DegU in controlling multicellular behaviour. Microbiology 155: 1-8. doi:10.1099/mic.0.023903-0. PubMed: 19118340. 19118340

[11] Kobayashi K (2007) Gradual activation of the response regulator DegU controls serial expression of genes for flagellum formation and biofilm formation in *Bacillus* *subtilis* . Mol Microbiol 66: 395-409. doi:10.1111/j.1365-2958.2007.05923.x. PubMed: 17850253. 17850253

[12] Verhamme DT, Kiley TB, Stanley-Wall NR (2007) DegU co-ordinates multicellular behaviour exhibited by *Bacillus* *subtilis* . Mol Microbiol 65: 554-568. doi:10.1111/j.1365-2958.2007.05810.x. PubMed: 17590234. 17590234

[13] Msadek T, Kunst F, Henner D, Klier A, Rapoport G et al. (1990) Signal transduction pathway controlling synthesis of a class of degradative enzymes in *Bacillus* *subtilis*: expression of the regulatory genes and analysis of mutations in *degS* and *degU* . J Bacteriol 172: 824-834. PubMed: 1688843. 1688843 10.1128/jb.172.2.824-834.1990 PMC208512

[14] Tokunaga T, Rashid MH, Kuroda A, Sekiguchi J (1994) Effect of DegS-DegU mutations on the expression of *sigD*, encoding an alternative sigma-factor, and autolysin operon of *Bacillus* *subtilis* . J Bacteriol 176: 5177-5180. PubMed: 7914190. 7914190 10.1128/jb.176.16.5177-5180.1994 PMC196365

[15] Mäder U, Antelmann H, Buder T, Dahl MK, Hecker M et al. (2002) *Bacillus* *subtilis* functional genomics: genome-wide analysis of the DegS-DegU regulon by transcriptomics and proteomics. Mol Genet Genomics 268: 455-467. doi:10.1007/s00438-002-0774-2. PubMed: 12471443. 12471443

[16] Amati G, Bisicchia P, Galizzi A (2004) DegU-P represses expression of the motility *fla-che* operon in *Bacillus* *subtilis* . J Bacteriol 186: 6003-6014. doi:10.1128/JB.186.18.6003-6014.2004. PubMed: 15342569. 15342569 PMC515139

[17] Kunst F, Pascal M, Lepesant-Kejzlarova J, Lepesant JA, Billault A et al. (1974) Pleiotropic mutations affecting sporulation conditions and the syntheses of extracellular enzymes in *Bacillus* *subtilis* 168. Biochimie 56: 1481-1489. PubMed: 4219582. 4219582 10.1016/s0300-9084(75)80270-7

[18] Henner DJ, Yang M, Ferrari E (1988) Localization of *Bacillus* *subtilis* *sacU*(Hy) mutations to two linked genes with similarities to the conserved procaryotic family of two-component signalling systems. J Bacteriol 170: 5102-5109. PubMed: 3141378. 3141378 10.1128/jb.170.11.5102-5109.1988 PMC211577

[19] Dahl MK, Msadek T, Kunst F, Rapoport G (1992) The phosphorylation state of the DegU response regulator acts as a molecular switch allowing either degradative enzyme synthesis or expression of genetic competence in *Bacillus* *subtilis* . J Biol Chem 267: 14509-14514. PubMed: 1321152. 1321152

[20] Amory A, Kunst F, Aubert E, Klier A, Rapoport G (1987) Characterization of the *sacQ* genes from *Bacillus* *licheniformis* and *Bacillus* *subtilis* . J Bacteriol 169: 324-333. PubMed: 3098732. 3098732 10.1128/jb.169.1.324-333.1987 PMC211771

[21] Msadek T, Kunst F, Klier A, Rapoport G (1991) DegS-DegU and ComP-ComA modulator-effector pairs control expression of the *Bacillus* *subtilis* pleiotropic regulatory gene *degQ* . J Bacteriol 173: 2366-2377. PubMed: 1901055. 1901055 10.1128/jb.173.7.2366-2377.1991 PMC207789

[22] Nagami Y, Tanaka T (1986) Molecular cloning and nucleotide sequence of a DNA fragment from *Bacillus* *natto* that enhances production of extracellular proteases and levansucrase in *Bacillus* *subtilis* . J Bacteriol 166: 20-28. PubMed: 3082853. 3082853 10.1128/jb.166.1.20-28.1986 PMC214550

[23] Mukai K, Kawata-Mukai M, Tanaka T (1992) Stabilization of phosphorylated *Bacillus* *subtilis* DegU by DegR. J Bacteriol 174: 7954-7962. PubMed: 1459944. 1459944 10.1128/jb.174.24.7954-7962.1992 PMC207531

[24] Ogura M, Tsukahara K (2012) SwrA regulates assembly of *Bacillus* *subtilis* DegU via its interaction with N-terminal domain of DegU. J Biochem 151: 643-655. doi:10.1093/jb/mvs036. PubMed: 22496484. 22496484

[25] Arnaud M, Chastanet M, Débarbouillé M (2004) New vector for efficient allelic replacement in naturally nontransformable, low-GC-content, gram-positive bacteria. Appl Environ Microbiol 70: 6887-6891. doi:10.1128/AEM.70.11.6887-6891.2004. PubMed: 15528558. 15528558 PMC525206

[26] Osera C, Amati G, Calvio C, Galizzi A (2009) SwrAA activates poly-gamma-glutamate synthesis in addition to swarming in *Bacillus* *subtilis* . Microbiology 155: 2282-2287. doi:10.1099/mic.0.026435-0. PubMed: 19389763. 19389763

[27] Calvio C, Celandroni F, Ghelardi E, Amati G, Salvetti S et al. (2005) Swarming differentiation and swimming motility in *Bacillus* *subtilis* are controlled by *swrA*, a newly identified dicistronic operon. J Bacteriol 187: 5356-5366. doi:10.1128/JB.187.15.5356-5366.2005. PubMed: 16030230. 16030230 PMC1196031

[28] Carey MF, Peterson CL, Smale ST (2012) Experimental strategies for the identification of DNA-binding proteins. Cold Spring Harb Protoc, 2012: 18–33. doi:10.1101/pdb.top067470. PubMed: 22194258. 22194258

[29] Tanaka T, Kawata M, Mukai K (1991) Altered phosphorylation of *Bacillus* *subtilis* DegU caused by single amino acid changes in DegS. J Bacteriol 173: 5507-5515. PubMed: 1909319. 1909319 10.1128/jb.173.17.5507-5515.1991 PMC208264

[30] Patrick JE, Kearns DB (2009) Laboratory strains of *Bacillus* *subtilis* do not exhibit swarming motility. J Bacteriol 191: 7129-7133. doi:10.1128/JB.00905-09. PubMed: 19749039. 19749039 PMC2772471

[31] Cairns LS, Marlow VL, Bissett E, Ostrowski A, Stanley-Wall NR (2013) A mechanical signal transmitted by the flagellum controls signalling in *Bacillus* *subtilis* . Mol Microbiol 90: 6-21. PubMed: 23888912. 23888912 10.1111/mmi.12342 PMC3963450

[32] Buelow DR, Raivio TL (2010) Three (and more) component regulatory systems - auxiliary regulators of bacterial histidine kinases. Mol Microbiol 75: 547-566. PubMed: 19943903. 19943903 10.1111/j.1365-2958.2009.06982.x

[33] Gordon AJ, Satory D, Halliday JA, Herman C (2013) Heritable change caused by transient transcription errors. PLoS Genet 9: e1003595. doi:10.1371/journal.pgen.1003595. PubMed: 23825966. 23825966 PMC3694819

