# Supplementary figures and images for: The Role of SwrA, DegU and PD3 in fla/che Expression in B. subtilis
Source: PLoS One. 2013 Dec 27;8(12):e85065. doi: 10.1371/journal.pone.0085065 (PMC3874003; doi:10.1371/journal.pone.0085065)

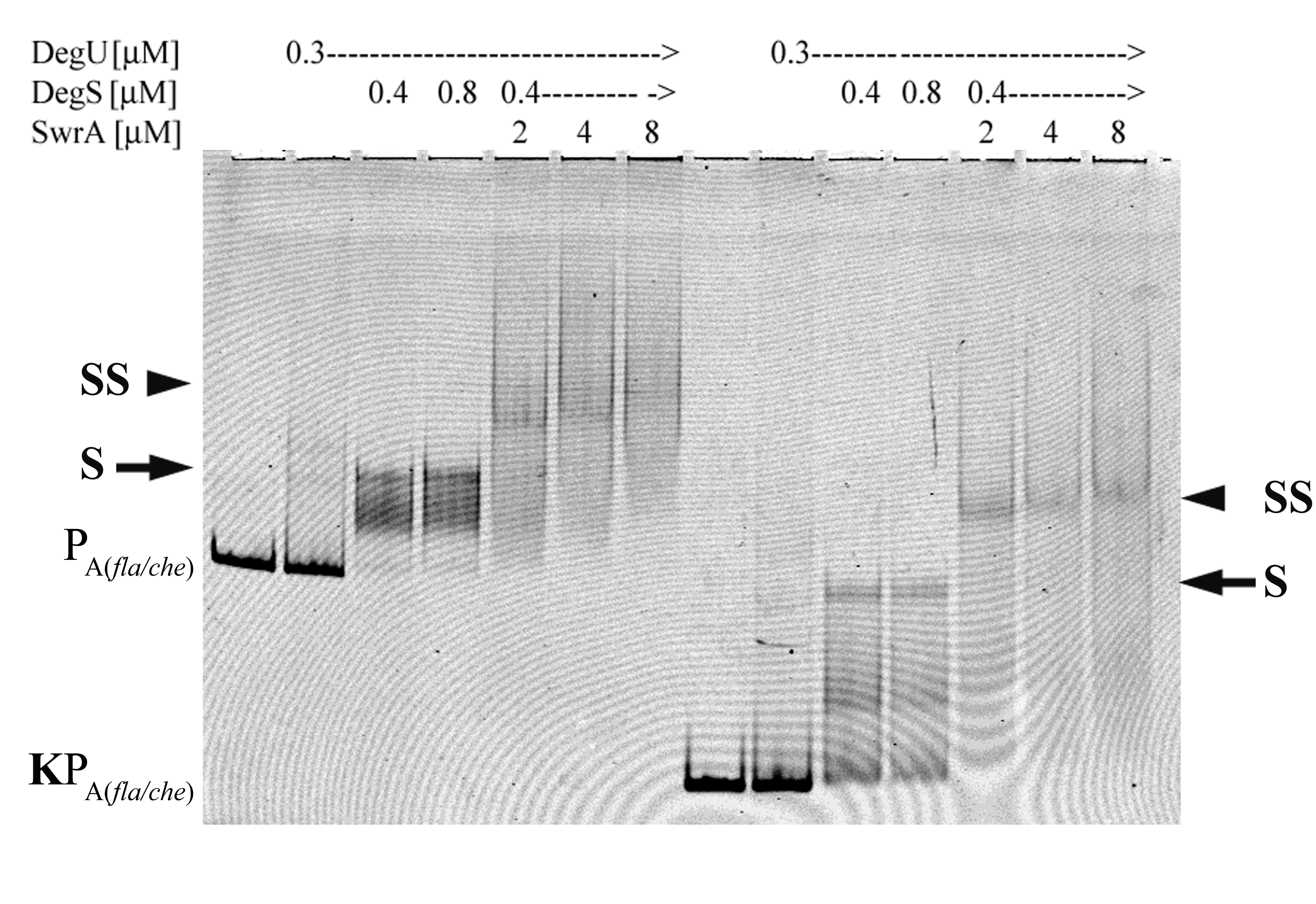

Supplement: Figure S1 — The S and SS complexes assemble also on a shorter probe K. EMSA were performed as described in Material and Methods with FAM-labeled probes. The final concentration (μM) of the proteins present in each reaction is indicated on top of the figure. In the first seven lanes reactions were performed with the standard PA(fla/che) probe used in Figures 2 and 4 of the manuscript; in the following seven lanes the shorter 156bp-probe K was used (depicted in Figure 2A). Probe K was amplified with FAM-labeled primer pair 8601 and 8756 (Table S3). (TIF) [file pone.0085065.s001.tif]

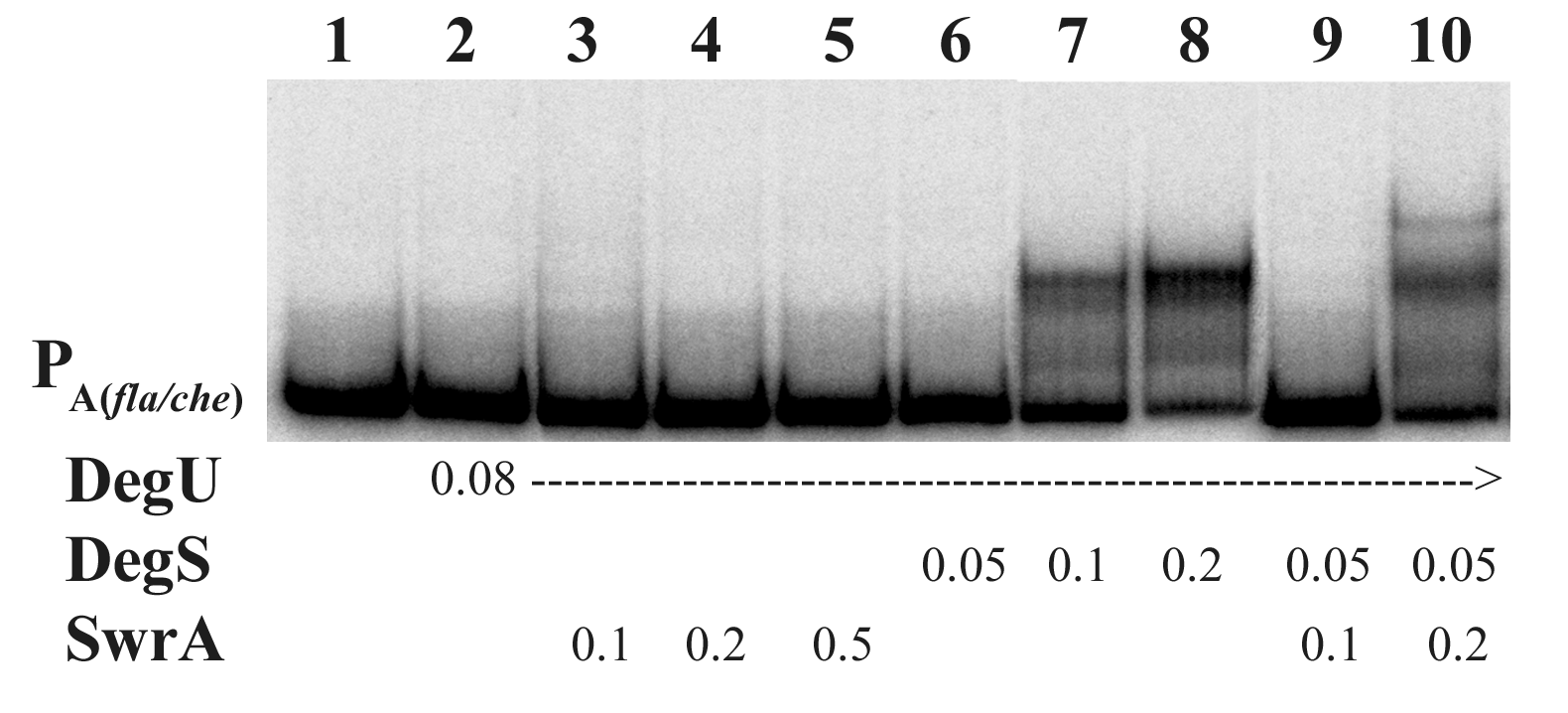

Supplement: Figure S2 — SwrA increases the DNA binding affinity of DegU~P. EMSA were performed with the standard radioactively-labeled PA(fla/che) as described in Material and Methods. DegU, DegS and SwrA were used in limiting concentrations as specified below each lane (final μM concentration in each reactions). The increase in affinity is evident by comparing lanes 6 and 10. (TIF) [file pone.0085065.s002.tif]

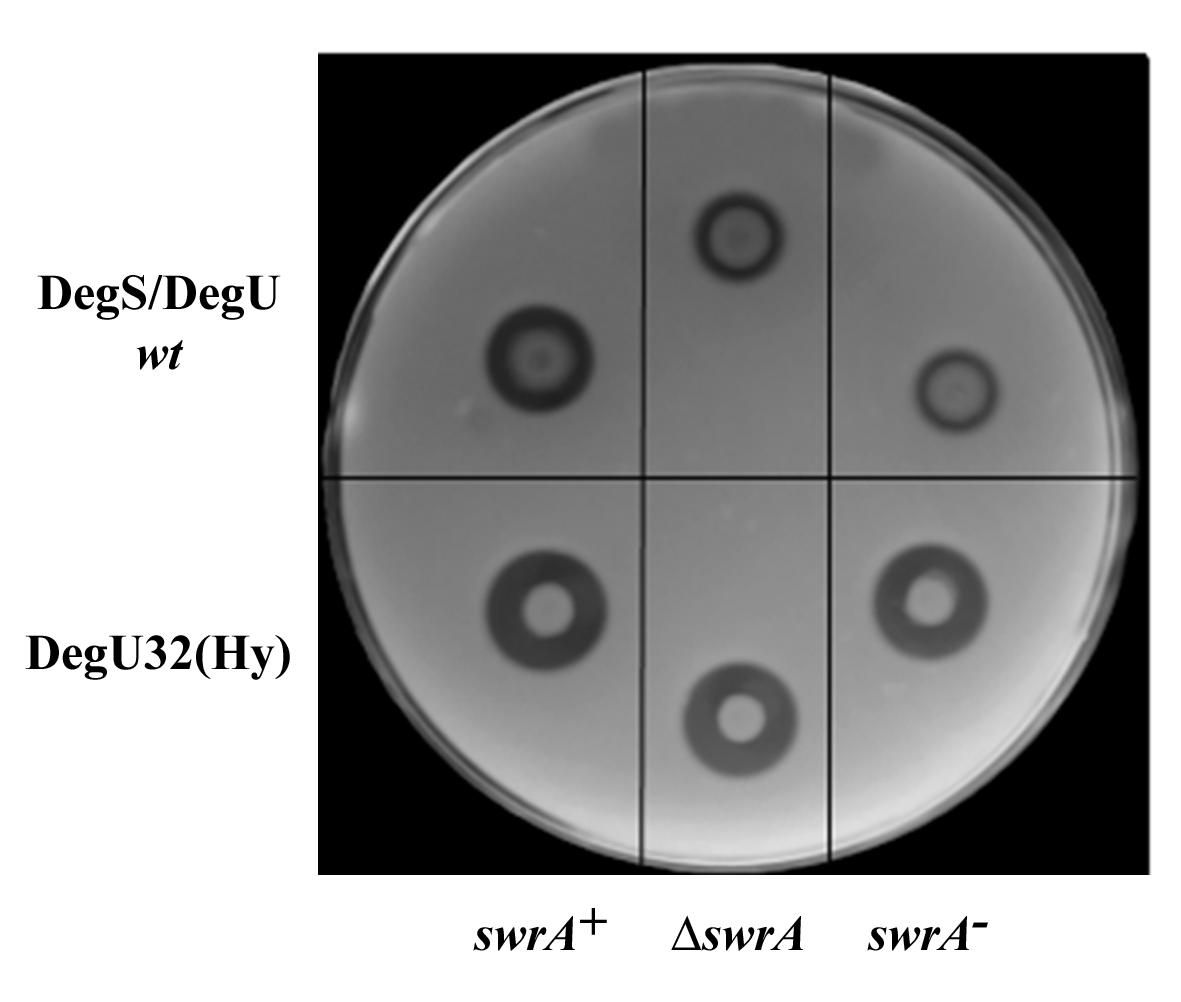

Supplement: Figure S3 — Extracellular protease secretion is enhanced by SwrA. Extracellular protease production was evaluated as previously described [12] by means of skim milk plates. DegU32(Hy) induces proteases secretion in either swrA + and swrA - isogenic strains as already published [13,17,19]. In a wild-type degS/degU background, though, also the SwrA- containing strain produces a higher level of proteases compared to the isogenic swrA - strain. Strains used to generate this image are: PB5249, PB5370, PB5383, PB5384 whose genotypes are listed in Table S1. The control strain carrying the swrA deletion (PB5334) has been previously described [27]. (TIF) [file pone.0085065.s003.tif]
