# Supplementary material for: The Role of SwrA, DegU and PD3 in fla/che Expression in B. subtilis
Source: PLoS One. 2013 Dec 27;8(12):e85065. doi: 10.1371/journal.pone.0085065 (PMC3874003; doi:10.1371/journal.pone.0085065)
Supplement: Table S1 — Strains used in this study. (DOCX) [file pone.0085065.s004.docx]

| **Strain** | **Relevant genotype** | **Source or reference** |
| --- | --- | --- |
| PB5249 | *trpC2 pheA1 swrA^+^* | (Senesi *et al.*, 2004) |
| PB5370 | *trpC2 pheA1 swrA^-^* | (Calvio *et al.*, 2008) |
| PB5383 | *trpC2 pheA1 swrA^+^ degU32*(Hy), Sp | (Osera *et al.*, 2009) |
| PB5384 | *trpC2 pheA1 swrA^-^ degU32*(Hy), Sp | (Osera *et al.*, 2009) |
| PB5390 | *trpC2 pheA1 swrA^+^ degS200*(Hy), Sp | (Osera *et al.*, 2009) |
| PB5391 | *trpC2 pheA1 swrA^-^ degS200*(Hy), Sp | (Osera *et al.*, 2009) |
| PB5392 | *trpC2 pheA1* P*_swrA_*WT- *swrA^+^,* Km | (Calvio *et al.*, 2008) |
| PB5394 | *trpC2 pheA1* P*_swrA_*D^-^-*swrA^+^,* Km | (Calvio *et al.*, 2008) |
| PB5396 | *trpC2 pheA1* P*_swrA_*WT-*swrA^-^,* Km | (Calvio *et al.*, 2008) |
| PB5427 | *trpC2 pheA1 pks::*P*_swrA_*WT-*lacZ* Δ*sigD,* Cm Em | (Calvio *et al.*, 2008) |
| PB5447 | *trpC2 pheA1 swrA^-^ dhsA6 degU32*(Hy), Sp | (Osera *et al.*, 2009) |
| PB5452 | *trpC2 pheA1 swrA^-^ dhsA6* | pMADdhs → PB5370 |
| PB5455 | *trpC2 pheA1* P*_swrA_*WT-*swrA^+^* ΔP_D3_*_(fla/che_*_)_, Km | pMADΔPD3 → PB5392 |
| PB5458 | *trpC2 pheA1* P*_swrA_*D^-^-*swrA^+^* ΔP_D3_*_(fla/che)_,* Km | pMADΔPD3→ PB5394 |
| PB5466 | *trpC2 pheA1* P*_swrA_*WT-*swrA^-^* ΔP_D3_*_(fla/che)_,* Km | pMADΔPD3 → PB5396 |

**Table S1. Strains used in this study**
