# Supplementary material for: The Role of SwrA, DegU and PD3 in fla/che Expression in B. subtilis
Source: PLoS One. 2013 Dec 27;8(12):e85065. doi: 10.1371/journal.pone.0085065 (PMC3874003; doi:10.1371/journal.pone.0085065)
Supplement: Table S2 — Plasmids used in this study. (DOCX) [file pone.0085065.s005.docx]

Mordini et al. “The role of SwrA, DegU and P_D3_ in *fla/che* expression in *B. subtilis”*

**Table S2. Plasmids used in this study**

| **Plasmid** | **Description^a^** | **Source or reference** |
| --- | --- | --- |
| pMADdhs | pMAD, *dhsA6*; *bgaB*; Em^r^ | Osera *et al.,* 2009 |
| pMADΔPD3 | pMAD, ΔPD3_(_*_fla/che_*_)_; *bgaB*; Em^r^ | This study |
| pET16Uwt | pET16b, *degU*; Am^r^ | Amati *et al.,* 2004 |
| pET16Uhy | pET16b, *degU32*(Hy); Am^r^ | Amati *et al.*, 2004 |
| pETOPO-DegSHy | pET101/D-TOPO, *degS200*(Hy); Am^r^ | This study |
| pETOPO-DegS | pET101/D-TOPO, *degS*; Am^r^ | This study |
| pGST-SwrA | pGEX-6P, *swrA*, Am^r^ | Calvio *et al*., 2005 |

***a***

^a^Abbreviations: Am^r^ ampicillin resistance; Em^r^ Erythromycin resistance.
