# Supplementary material for: The Role of SwrA, DegU and PD3 in fla/che Expression in B. subtilis
Source: PLoS One. 2013 Dec 27;8(12):e85065. doi: 10.1371/journal.pone.0085065 (PMC3874003; doi:10.1371/journal.pone.0085065)
Supplement: Table S3 — Primers used in this study. (DOCX) [file pone.0085065.s006.docx]

**Table S3. Primers used in this study**

| **Primer** | **Sequence** (restriction sites are underlined) |
| --- | --- |
| CodYF (*Eco*RI) | 5’-CGGAATTCTAGTGAATATACTGCTTTCCC-3’ |
| nRflgBN (*Nco*I) | 5’-GCCCATGGTTATAGTTCGGTGTATCTATATTG-3’ |
| 8601 | 5’-CGAGAAATGTAGTTCTAACAATC-3’ |
| 8465 | 5’-CAACCCGAGAAATAAAGC-3’ |
| cs8610 | 5’-AGTTCTAACAATCTAGGAC-3’ |
| 8756 | 5’-ACCCTCAATATCCTTGTCGAG-3’ |
| DegSFcacc | 5’-CACCATGAATAAAACAAAGATGGATTCCAA-3’ |
| DegSRblunt | 5’-AAGAGATAACGGAACCTTAATCAT-3’ |
| R3 | 5’-GGAGGATCCCGCTTCCTTCAGGTCGAACC-3’ |
| S3 | 5’-GGCCTGATTCCAACTTTAAG-3’ |
